# Urban compactness and Land Use Efficiency in Indochina Capitals: A multi-method spatial assessment (2017–2024)

**DOI:** 10.1371/journal.pone.0350805

**Published:** 2026-06-05

**Authors:** Tran Tuan Nguyen

**Affiliations:** Faculty of Real Estate and Resources Economics, College of Business, National Economics University, Hanoi, Vietnam; Military University of Technology Faculty of Civil Engineering and Geodesy: Wojskowa Akademia Techniczna im Jaroslawa Dabrowskiego Wydzial Inzynierii Ladowej i Geodezji, POLAND

## Abstract

This study assesses land use efficiency (LUE) and urban spatial structure in three Indochina capitals, Hanoi, Vientiane, and Phnom Penh, during the period 2017–2024. By integrating the SDG 11.3.1 indicators with spatial agglomeration analysis (global Moran’s I) and urban form measurement (Polsby-Popper index), the study provides a multidimensional assessment of urban expansion patterns. The results indicate that land expansion outpaces population growth in all three cities (LUE > 1). Phnom Penh exhibited the highest LUE value (5.03), strong spatial agglomeration, and the most compact urban form. Hanoi recorded the lowest LUE (2.47), characterized by moderate compactness and weak spatial autocorrelation. In contrast, Vientiane displayed fragmented and largely random urban expansion, accompanied by a declining compactness index and no statistically significant agglomeration. These contrasting patterns reflect distinct urban development trajectories shaped by differences in planning regimes, land management practices, and investment orientations. The findings highlight the urgent need for spatially controlled and compact urban development strategies in rapidly growing cities, while also acknowledging data limitations. The proposed analytical framework is transferable and can be applied to other developing cities worldwide.

## 1. Introduction

Rapid urbanization is profoundly transforming the spatial structure of cities worldwide [1,2], especially in developing countries [3,4], where the rate of land conversion often outpaces population growth [5]. In this context, urban LUE has emerged as a crucial indicator for assessing the level of sustainable urban development [6]. The importance of LUE is underscored by the United Nations Sustainable Development Goal (SDG), “Sustainable Cities and Communities”. LUE, as operationalized under SDG indicator 11.3.1, is defined as the ratio between the land consumption rate (LCR) and the population growth rate (PGR) [6]. It provides a quantitative measure of the extent to which urban land expansion is aligned with demographic growth. A value close to 1 indicates a balanced relationship between land consumption and population growth, suggesting relatively compact and efficient urban development. In contrast, values greater than 1 imply that land is being consumed faster than population increase, often reflecting urban sprawl and inefficient spatial expansion, while values below 1 indicate densification or population concentration processes [7]. However, sustainable urban development depends not only on the balance between land and population but also on how urban space is organized and the city’s physical form [8].

Beyond indicator-based assessments, recent urban theories have emphasized the importance of balanced and equitable spatial development. One such perspective is isobenefit urbanism, which advocates that urban areas should provide relatively equal access to urban benefit, including green spaces, services, and infrastructure, across locations [9,10]. This approach promotes a balanced relationship between built and non-built environments and encourages multifunctional land use patterns. In this sense, LUE is not only a measure of proportionality between LCR and PGR, but also relates to broader notions of spatial balance, accessibility, and equity in urban development [11]. Spatial autocorrelation indices such as Global Moran’s I help reveal whether areas with high or low land use efficiency are clustered or dispersed across a city [12,13]. A significant Moran’s I value indicates that similar efficiency levels (either high or low) tend to group together geographically, offering insights into the underlying urban structure. In parallel, geometric compactness indices such as Polsby-Popper index quantify the shape of urban expansion by comparing a city’s footprint to an ideal compact shape [14]. Lower Polsby-Popper values signal more elongated or irregular urban boundaries, characteristic of sprawling development. Examining both the spatial distribution of efficiency and the compactness of urban form provides a deeper understanding of urban growth dynamics [15].

The Indochina region illustrates these concepts through the divergent development patterns of its three capital cities, Hanoi (Vietnam), Phnom Penh (Cambodia), and Vientiane (Laos). Despite close geographical proximity and some shared historical contexts, each city is at a distinct stage of urban development and has followed a different trajectory of urban expansion. These differences stem from varying institutional frameworks, infrastructure investments, and planning capacities unique to each city. Previous studies in the region have contributed important insights into urban expansion [16–18], yet they exhibit several key limitations. First, most analyses focus on land consumption dynamics or urban sprawl indicators without explicitly linking land expansion to population growth within the SDG 11.3.1 framework [19,20]. As a result, land use efficiency is often assessed independently of spatial structure. Second, studies that apply spatial autocorrelation measures typically examine built-up density or land cover patterns, rather than the spatial distribution of efficiency itself [21,22]. These limits are understanding of whether efficient or inefficient land use tends to concentrate or disperse within urban areas. Third, urban form indicators such as geometric compactness are analyzed as descriptive shape metrics, without being systematically connected to land-population balance or spatial clustering process [23,24].

Consequently, existing research rarely captures the joint interaction between land use efficiency, spatial organization, and urban morphology. This separation obscures how cities simultaneously balance land consumption with population growth, organize development spatially, and shape compact or fragmented urban forms. The lack of an integrated analytical framework is particularly evident in comparative studies of medium-sized and rapidly growing capital cities in Indochina, where different planning regimes and governance capacities may produce distinct but understudied spatial outcomes. To address this gap, the present study proposes a novel comparative framework that combines all three aspects of urban development, including land use efficiency, spatial autocorrelation, and geometric compactness. This study assesses and compares the urban LUE, the spatial patterns of LUE (via Global Moran’s I), and the compactness of urban form (via the Polsby-Poper index) for Hanoi, Phnom Penh, and Vientiane over the period 2017–2024. Using high-resolution ESRI datasets (10 m) and WorldPop population data were employed to calculate the land consumption rate (LCR), population growth rate (PGR), and LUE at the grid level, following the SDG 11.3.1 framework. By integrating these metrics, the analysis offers a comprehensive view of urban expansion. It reveals not only how efficiently each city is using land relative to its population growth, but also where efficient or inefficient growth is occurring and how contiguous or fragmented each city’s expansion has become. The findings offer empirical evidence for the spatial urban efficiency approach, contributing to policy formulation aimed at promoting more compact and sustainable urban development in the rapidly transforming Southeast Asian context.

## 2. Study area

**Fig 1:**
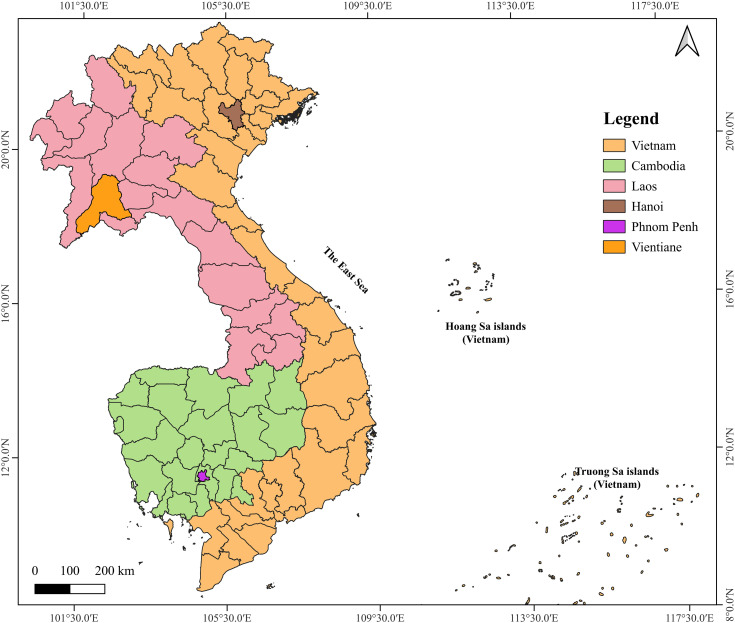
Study areas. Administrative boundaries obtained from the geoBoundaries. **Source:** author’s compilation, 2025.

The Indochina Peninsula is in mainland Southeast Asia and has three capital cities: Hanoi (Vietnam), Phnom Penh (Cambodia), and Vientiane (Laos) (Fig 1). While these cities share certain geographic and historical features, they represent markedly different trajectories of urban development. Geographically, Hanoi is located in northern Vietnam, within the fertile Red River Delta, bordered by the Red River to the east and the Da River to the west [17]. Phnom Penh lies in south-central Cambodia, at the confluence of three major rivers converge: the Mekong, the Tonlé Sap, and the Bassac [25]. Vientiane is situated in central Laos, on the left bank of the Mekong River, directly adjacent to the Thai border. For all three cities, this study uses administrative boundaries to define the urban extent. In the case of Vientiane, the study acknowledges that its proximity to the Thai border and cross-border linkages with Nong Khai create a larger functional urban area. However, for consistency in national comparisons and data availability, the study limits the analysis to the administrative area of Vientiane. Demographically, Hanoi is the largest of the three capitals, with an estimated population of 8.69 million in 2024, representing 8.6% of Vietnam’s national population [26]. Its population grew at an average annual rate of 2.22% between 2009 and 2019, although this rate has shown signs of deceleration in the early 2020s [26]. Phnom Penh ranks second in population size but has the fastest growth rate, estimated at 2.4 million people in 2024 and an average annual growth of approximately 3.9% in recent year [27]. Vientiane remains the smallest capitals, with an estimated 738,000 residents in 2024, representing roughly 11% of Laos’s national population. Its population growth rate has remained moderate, averaging around 2% per year [28].

Economically, Hanoi functions as Vietnam’s political and economic hub, with a robust growth rate ranging from 6% to 9% annually in the early 2020s [17]. It continues to attract investment and industrial development. Phnom Penh has also experienced rapid economic expansion, driven largely by foreign direct investment, particularly from China, as well as growth in the garment, real estate and service sectors [29]. Vientiane’s economy remains the smallest and most modest among the three, growing at 4.83% in 2022 [30]. Population density varies significantly due to differences in administrative area and urban form. Phnom Penh has the highest density, with approximately 5,700 inhabitants per km^2^ across a 232 km^2^ area [27]. Hanoi’s density is lower due to its much larger administrative area (3,359 km^2^), while Vientiane is the least dense, averaging about 188 inhabitants per km^2^ [28]. These geographic, demographic, and economic contrasts reflect the distinct urban dynamics shaping each capital. They also underscore the importance of analyzing urban LUE across different spatial, institutional, and developmental contexts.

## 3. Data collection and methods

This study employs spatial datasets from global open sources to analyze land use and population dynamics in the three Indochinese capitals by QGIS 3.44.1. Built-up land information was derived from the ESRI Global Land Use/Land Cover dataset with a spatial resolution of 10 m for the years 2017 and 2024, enabling precise detection of urban expansion. Population data were obtained from the WorldPop project, which provides gridded population estimates at a 100 m resolution, standardized by administrative boundaries. Administrative boundary shapefiles for Hanoi, Phnom Penh, and Vientiane were sourced from the geoBoundaries database. To ensure spatial consistency, all datasets were projected to the Universal Transverse Mercator (UTM) coordinate reference system. To enable consistent spatial comparison across the three cities, each study area was divided into 500 m grid cells. This resolution was selected to balance spatial detail with the resolution of input data and to reduce scale-related bias. Each grid cell aggregates approximately 2,500 pixels from the 10m land cover data and 25 pixels from the 100 m population data, ensuring sufficient detail while maintaining statistical robustness. The 500 m scale also helps mitigate the Modifiable Areal Unit Problem and has been widely used in previous intra-urban studies for land use efficiency analysis [31,32]. The ESRI land use raster was reclassified to retain only the built-up land category, which served as a proxy for urbanized land. The original LULC product includes nine thematic classes: water, crops, snow/ice, trees, built area, clouds, flooded vegetation, bare ground, and rangeland. For the analysis, only the built area class was retained, while all other classes were group as “non-built” and excluded from further calculation. No additional reclassification or manual editing was applied. Pixel labeled as “Cloud” or “No data” were treated as missing and masked out during the spatial overlay. WorldPop population data for 2017 and 2024 were also combined at the grid-cell level so that the distribution and density of the population could be compared over time.

To quantify the relationship between spatial expansion and population growth, the LUE index was computed following the standard methodology of SDG indicator 11.3.1, which compares the land consumption rate (LCR) and the population growth rate (PGR) over a defined period. The LUE was evaluated at two spatial levels: the total urban area and separate grid cells.

Although the official SDG 11.3.1 methodological framework recommends a linear formulation for LCR and a logarithmic formulation for PGR, this study adopts a logarithmic function for both variables. This adjustment ensures mathematical consistency between the numerator and denominator in the LUE ratio and has been widely implemented in recent academic studies [33–35]. The log-log adjustment ensures dimensional consistency between the numerator and denominator in the LUE ratio, thereby avoiding interpretive distortions when comparing growth rates expressed in different functional forms. Moreover, the log-log approach reduces the influence the extreme values and outliers, which is particularly important in rapidly growing or shrinking urban areas where raw area changes can be volatile. More importantly, several recent studies have shown that urban land and population tend to follow allometric or power-law relationships, especially at the intra-urban or grid-cell scale, where density patterns are uneven [36,37]. By applying logarithmic functions to both built-up area and population, the model captures elasticity directly and facilitates comparability across cities of different sizes and growth trajectories. This approach has been used in recent global assessments of SDG 11.3.1, where logarithmic transformations enable scale-neutral comparisons of urban expansion [38,39].

At the urban scale, the LUE index was computed based on the aggregate built-up land area and population encompassed by the administrative boundaries of each capital city, as formulated in Equation (1):


$$\begin{array}{c} LUE=\;\frac{LCR}{PGR}=\frac{\text{ln}\left({BU}_{\text{2}\text{0}\text{2}\text{4}}\right)-\text{ln}\left({BU}_{\text{2}\text{0}\text{1}\text{7}}\right)}{\text{ln}\left({Pop}_{\text{2}\text{0}\text{2}\text{4}}\right)-\text{ln}\left({Pop}_{\text{2}\text{0}\text{1}\text{7}}\right)}\; \end{array}$$ (1)

where $BU_{t}$denotes the total built-up area (m²) within the administrative boundary of the city in year *t*, and $Pop_{t}$represents the corresponding total population in the same year.

At the grid level, LUE was calculated separately for each 500 m × 500 m cell to capture spatial variations in land use dynamics. To mitigate potential log (0) computational issues, a small constant (ε) was introduced to all variables. The value of ε was set to 1% of the grid area, equivalent to 2,500 m² in the LCR calculation, and 10 persons in the PGR calculation. The selection of 2,500 m^2^ as a threshold is applied by many land-use mapping programs to identify significant urban areas [40]. Hence, the threshold of 2,500 m^2^, representing 1% of a 500m x 500m grid cell, was chosen as a theoretically grounded and empirically reasonable minimum footprint. The LUE formula applied to each grid cell is presented in Equation (2).


$$\begin{array}{c} {LUE}_{i}=\frac{{LCR}_{i}}{{PGR}_{i}}=\frac{\text{ln}\left({BU}_{i,\;\text{2}\text{0}\text{2}\text{4}}+\varepsilon_{\text{1}}\right)-\text{ln}\left({BU}_{i,\;\text{2}\text{0}\text{1}\text{7}}+\varepsilon_{\text{1}}\right)}{\text{ln}\left({Pop}_{i,\text{2}\text{0}\text{2}\text{4}}+\varepsilon_{\text{2}}\right)-\text{ln}\left({Pop}_{i,\text{2}\text{0}\text{1}\text{7}}+\varepsilon_{\text{2}}\right)}\; \end{array}$$ (2)

where $BU_{i,t}$denotes the total built-up area (m²) contained within the *i*th grid cell in year *t*, and $Pop_{i,t}$represents the corresponding total population of that grid cell in the same year.

To examine the spatial structure of land use efficiency, the Global Moran’s I index was computed based on the gridded LUE values using a row-standardized spatial weights matrix. The objective of this analysis is to identify whether high or low LUE values exhibit spatial clustering or spatial dispersion. The Global Moran’s I statistic is formulated in Equation (3).


$$\begin{array}{c} I=\frac{n}{W}\frac{\sum_{i=\text{1}}^{n}\sum_{j=\text{1}}^{n}w_{ij}\left(x_{i}-\overset{―}{x}\right)\left(x_{j}-\overset{―}{x}\right)}{\sum_{i=\text{1}}^{n}{\left(x_{i}-\overset{―}{x}\right)}^{\text{2}}} \end{array}$$ (3)

where *n* is the number of grid cells, *xᵢ* represents the LUE value of the *i*th cell, $\bar{x}$ denotes the mean LUE value across all cells, *wᵢⱼ* indicates the spatial weight between cells *i* and *j*, and *W* refers to the total sum of spatial weights in the matrix.

To evaluate the morphological compactness of urban areas, the study employed the Polsby–Popper compactness index (PP), formulated in Equation (4).


$$\begin{array}{c} PP=\frac{\text{4}\pi A}{P^{\text{2}}} \end{array}$$ (4)

where A denotes the built-up area and P represents the perimeter of the contiguous urban boundary obtained by merging individual built-up polygons. A higher PP value signifies a more compact and regular urban structure, whereas a lower value denotes a more dispersed and irregular spatial configuration.

## 4. Results

### 4.1. Comparative analysis of land use efficiency

At the urban level, the LUE index exhibits clear differences among the three Indochinese capitals (Table 1). Phnom Penh recorded the highest LUE (5.03), followed by Vientiane (3.65), while Hanoi showed the lowest value (2.47). This ranking reflects substantial variation in the relationship between urban land expansion and population growth across the three cities. Phnom Penh’s high LUE primarily results from a very rapid rate of land consumption (LCR = 49.22), which far exceeds its population growth rate (PGR = 9.79). This imbalance indicates a pronounced trend of urban sprawl, where built-up areas expand much faster than population demand. By contrast, Hanoi demonstrates a more balanced development pattern, with a relatively low land consumption rate (LCR = 15.84) and a moderate population growth rate (PGR = 6.41), suggesting more efficient use of urban land per unit of population growth. Vientiane lies between the two extremes: although it experienced the highest population growth rate (10.83), its land consumption rate is also high (39.50), resulting in a relatively elevated LUE value.

**Table 1:** City-wide land use efficiency comparison.

Categories	LCR	PGR	LUE
Hanoi	15.84	6.41	2.47
Vientiane	39.50	10.83	3.65
Phnom Penh	49.22	9.79	5.03

**Source:** author’s calculations, 2025.

**Fig 2:**
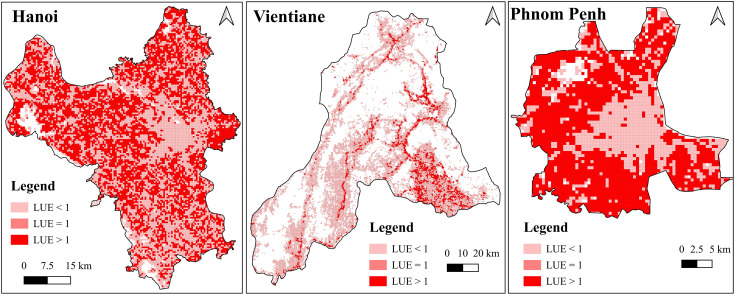
Spatial distribution of LUE at grid level using ESRI/ Impact Observatory Land Cover data (CC BY 4.0) and WorldPop population data. **Source:** author’s compilation, 2025.

The spatial distribution of LUE at the grid-cell level provides deeper insights into micro-scale land use dynamics within each urban area (Fig 2). In Hanoi, grid cells with LUE > 1 are widely distributed across the territory, particularly along the western and southern expansion corridors. This pattern indicates that in many zones, land consumption continues to outpace population growth. Conversely, the urban core and certain outer belts exhibit clusters of LUE < 1, suggesting compact development or population-driven infill processes. In Vientiane, most built-up areas display LUE > 1, with distinct hotspots concentrated along major radial traffic axes and in the southern sector. The dominance of high-LUE cells indicates that spatial expansion generally exceeds demographic growth across much of the study area. Only a few grid cells fall within the LUE ≤ 1 category, reflecting limited instances of compact or population-efficient land use. By contrast, Phnom Penh reveals a distinct spatial pattern. Both central and peripheral zones are largely characterized by LUE > 1, indicating extensive oversprawl. Unlike the more scattered distribution observed in Vientiane, high-LUE cells in Phnom Penh form contiguous clusters, particularly in the southern and western sectors of the city. Notably, a narrow strip of LUE < 1 cells emerges in the urban core, suggesting localized infill development or intensified population concentration.

### 4.2. Spatial autocorrelation of land use efficiency

The Global Moran’s I index was applied to the LUE values to assess the spatial association of land use efficiency among grid cells within each urban area. This index evaluates whether cells with similar values, either high or low, tend to form spatial clusters or are randomly distributed. The results reveal distinct spatial autocorrelation patterns across the three capitals (Table 2). Phnom Penh exhibits the strongest spatial convergence, with a Moran’s I value of 0.1237 and a very high z-score (16.07, p < 0.001). This indicates that high-LUE cells are not randomly distributed but are spatially clustered, suggesting a clear concentration of oversprawl areas. By comparison, Hanoi presents a markedly different pattern. Although its Moran’s I value is slightly positive (0.0006) and statistically significant (p = 0.0237), the degree of spatial autocorrelation is weak, implying that the spatial distribution of LUE is neither strongly clustered nor completely random. Unlike the two aforementioned cities, Vientiane exhibits no statistically significant spatial clustering, with a Moran’s I value close to zero (−0.00005), a z-score near zero (−0.00027), and an insignificant p-value (0.9998). This suggests a completely random spatial distribution of LUE values across grid cells, with no evident clustering or orderly dispersion. Overall, the contrasting spatial autocorrelation patterns highlight different urban growth dynamics and spatial planning efficiencies among the three Indochinese capitals.

**Table 2:** Moran’I Index of LUE.

Categories	Moran’I	Expected Index	z-score	p-value
Hanoi	0.000615	−0.000075	2.262227	0.023683
Vientiane	−0.000050	−0.000050	−0.000270	0.999785
Phnom Penh	0.123681	−0.000364	16.065398	0.000000

**Source:** author’s calculations, 2025.

### 4.3. Urban morphology assessment using Polsby-Popper index

To quantify the geometric characteristics of urban form, the Polsby-Popper (PP) index was applied to evaluate the compactness and spatial continuity of built-up areas (Table 3). Among the three capitals, Phnom Penh exhibited the highest level of compactness, with the PP value increasing from 0.00105 in 2017 to 0.00120 in 2024 (ΔPP = +0.00015). This increase indicates that despite substantial spatial expansion, the city’s urban form has become more cohesive and spatially integrated. In contrast, Hanoi recorded the lowest PP index in both periods, rising only marginally from 0.00013 to 0.000135 (ΔPP = +0.0000065). This negligible increase suggests that although built-up land has expanded, the overall urban structure remains highly fragmented. Vientiane exhibited an opposite trend, with the PP index declining from 0.000283 in 2017 to 0.000271 in 2024 (ΔPP = −0.000012). This reduction implies that the city’s morphology has become more irregular and spatially disconnected over time.

**Table 3:** Urban compactness (Polsby–Popper Index).

Categories	PP_index		ΔPP
**2017**		**2024**
Hanoi	0.00012857	0.00013511	0.00000654
Vientiane	0.00028351	0.00027147	−0.00001204
Phnom Penh	0.00104958	0.00120412	0.00015454

**Source:** author’s calculations, 2025.

## 5. Discussion

### 5.1. Interpretation of LUE results and comparison with global and regional literature

The results reveal that all three capital cities exhibit a LUE index above 1 during the period 2017–2024, indicating that urban land area has expanded several times faster than population growth (The LUE index in Hanoi, Vientiane, and Phnom Penh is 2.47, 3.65, and 5.03, respectively.). This pattern reflects a pronounced trend of urban sprawl, consistent with the broader context of rapidly developing cities. Phnom Penh’s exceptionally high LUE value was more than 5, indicating intense suburban expansion and extensive land consumption in the Cambodian capital. Hanoi, by contrast, exhibits the lowest LUE among the three cities, suggesting a somewhat more balanced relationship between land consumption and population growth, although its value above 1 still implies a prevailing tendency toward sprawl rather than compaction. This observation aligns with evidence from recent urbanization patterns in Vietnam, where the spatial expansion of built-up land has significantly outpaced population growth, leading to declining density and reduced urban productivity [41]. Overall, the LUE outcomes for Hanoi, Vientiane, and Phnom Penh during 2017–2024 reflect a shared trajectory of unsustainable urban development across Southeast Asia, where cities continue to expand spatially beyond the demographic demand that their populations generate.

Compared with previous studies on Southeast Asian and other developing cities worldwide, the findings of this study align with the widely documented trend of rapid suburbanization and low land use efficiency. For instance, Seto et al. (2011) [42] demonstrated that in most cities globally, urban land expansion outpaced population growth during 1970–2000. Similarly, the World Bank estimates that urban land area worldwide has expanded approximately 50% faster than urban population growth on average [43]. Extending this analysis, Matta et al. (2022) [44] identified more nuanced regional patterns between 1970 and 2014, noting that while population growth surpassed urban land expansion in Africa, the opposite occurred in India, Central and South America, China, East Asia, Southeast Asia, and Europe, where land consumption outstripped demographic change. Lou et al. (2025) [45] also showed similar results in East Asia and Southeast Asia. In this context, Vientiane and Phnom Penh exemplify urban sprawl dominated by land expansion rather than population growth, patterns similar to those observed in many Chinese cities [46,47], Spain [48]. With LUE values exceeding 3, both capitals exhibit a clearly unsustainable urbanization trajectory. This outcome mirrors trends reported in other medium-sized cities, where the growth of built-up land has far exceeded population increase, as several studies in Laos and Cambodia have cautioned. These comparative insights underscore the persistence of land-driven urbanization in emerging economies, emphasizing the urgent need for policies promoting compact, efficient, and sustainable spatial development.

### 5.2. Interpretation of Polsby-Poper compactness and Moran’s I

In addition to measuring the magnitude of urban expansion, this study also examined urban spatial form using the Polsby-Popper compactness index and spatial concentration through Moran’s I. These results highlight distinct differences in the urban expansion patterns of the three capitals. Although the absolute PP values vary with city size, the increasing trend observed in Hanoi and particularly in Phnom Penh suggests that their urban forms have become slightly more compact over time. By contrast, the decreasing PP in Vientiane indicates a more dispersed and less cohesive urban morphology. Uncontrolled urbanization on former agricultural land in Vientiane may have contributed to this dispersed pattern [49]. Between 2016 and 2020, the city’s built-up area tripled, largely through the conversion of agricultural land, a rapid spatial expansion relative to its small population base, likely resulting in a fragmented and inefficient urban structure [49]. Phnom Penh, on the other hand, demonstrates the opposite trend. Increasing PP values there reflect growing spatial compactness, possibly due to concentrated expansion along specific development corridors. Since around 2006, urban growth in Phnom Penh has largely extended toward natural lakes and peripheral wetlands, driven by the proliferation of large-scale real estate projects in these areas [25].

The Global Moran’s I analysis of LUE offers deeper insights into the spatial distribution of land use efficiency within each urban area. In Hanoi, and particularly in Phnom Penh, areas with high LUE values tend to be spatially proximate, creating a positive spatial correlation. This indicates a clear spatial concentration of urban expansion patterns. By contrast, Vientiane exhibits an almost random distribution of LUE values, with no discernible clusters of high- or low-efficiency areas. These differences reveal distinct modes of urban expansion and spatial organization among the three capitals. In Phnom Penh, the rapid concentration of large-scale development projects on wetlands near the urban core has produced intensive clusters of expansion [50], resulting in a high Moran’s I value. Such spatial growth contributes to an uneven distribution of urban benefits and environmental costs across settlements [25]. Similarly, the positive Moran’s I observed in Hanoi suggests several dominant development corridors, particularly toward the western and northern sectors, where new urban zones and ring roads have emerged [51]. This finding aligns with recent planning trends showing that Hanoi’s expansion has been concentrated along major highways and newly planned growth poles [17]. In contrast, urban expansion in Vientiane has occurred more diffusely, across small and scattered areas, without forming sufficiently large clusters to generate significant spatial correlation. This pattern reflects limited spatial planning and weak land management enforcement [52]. Previous studies have noted the absence of strict implementation of land use plans in Vientiane, which has encouraged spontaneous suburban settlements and uncontrolled spatial sprawl [49,53]. Consequently, the LUE values across grid cells in Vientiane exhibit minimal variation, resulting in an insignificant Moran’s I statistic.

### 5.3. Policy implications

Beyond the individual cities, the findings have broader implications for the region’s urban development and relevant policies, especially in the context of SDG 11.3.1. The presence of LUE values above 1 in the study signals that urban land in this region is being used less efficiently than ideal, echoing patterns observed in many developing areas. If cities in the region continue to consume land faster than their population grows, they risk undermining goals of sustainable urbanization. This is directly pertinent to SDG 11.3.1, which aims for cities to balance land consumption with population growth. At the regional scale, the findings highlight the need for coordinated urban planning policies. Hence, addressing the drivers, such as rapid economic growth, inward migration, and the proliferation of new industrial zones, requires policy responses not just at the city level but also regionally. For example, regional planning authorities could implement urban growth boundaries of land-use zoning that collectively guide cities toward compact growth strategies. Policies promoting higher-density development in city centers and discouraging uncontrolled suburban subdivision could be adopted across the region to align with SDG 11.3.1 objectives.

The results also carry implications for infrastructure and resource management. Inefficient land use (high LUE) tends to increase per-capita infrastructure costs, providing roads, transit, water, and electricity over sprawling areas is more expensive and less sustainable. It can also lead to the loss of agricultural land and green spaces, affecting regional food security and environmental health. Thus, improving LUE is not only about meeting a numeric target but also about achieving cost-effective, environmentally sound urban growth. Regional policymakers should integrate the findings into broader plans such as climate change adaptation and economic development strategies. Moreover, by linking directly to SDG 11.3.1, the study underscores the importance of localizing global goals. It demonstrates how regional data and analysis can inform whether a country or province is on track to make cities inclusive, resilient, and sustainable. The relatively inefficient land use in these case cities suggests that without changes, the region may struggle to meet SDG 11 targets by 2030. In sum, the broader implication is a call for policy action at multiple levels, local city authorities, regional planners, and national urban policymakers, to adopt measures that ensure land is used judiciously in tandem with population growth. This aligns with international recommendations for sustainable urban development and provides empirical evidence from our region to support those recommendations.

### 5.4. Study limitations and future research directions

While the study provides valuable insights, it is important to acknowledge several limitations that may affect the interpretation of the results. This study relied on the ESRI 10 m land cover dataset to delineate built-up areas. Although this dataset offers high resolution, it is not free from error. Classification uncertainties can lead to misidentification of urban extents, especially in heterogeneous landscapes. Besides, the population growth estimates come from WorldPop gridded data, which has limitations. WorldPop uses demographic and geospatial covariates to distribute population, and it may not capture actual population distribution within cities. The analysis is somewhat narrow in focus, examining LUE and spatial metrics, and does not explicitly include socioeconomics or policy factors that could be influencing the results. For instance, differences in governance, economic structure, or land management policies between cities are not quantitatively assessed, yet they likely play a role in why one city sprawls more than another. Similarly, environmental constraints were not factored into compact interpretation. This is acknowledged as a limitation and an area for complementary qualitative analysis.

Building on this study’s findings and acknowledging its limitations, there are several directions for future research. Expanding the scope beyond the current cities would provide a broader comparative framework. A multi-city or across-country study in the region could test whether the patterns the findings observed hold true generally. It would be insightful to include both large metropolitan areas and smaller town, that can be “do mega-cities exhibit fundamentally different LUE dynamics and spatial autocorrelation patterns compared to medium-sized cities?”. Another direction involves delving into qualitative and policy research to complement the quantitative metrics. Case studies of the cities through interviews, policy document analysis, and field observation could uncover the underlying causes of their land use patterns. Understanding decision-making, urban planning history, or cultural factors can explain why the numbers look as they do, offering lessons that pure data cannot. Future research could explore new metrics and models to extend this analysis. The Polsby-Poper index and Moran’s I are useful, but urban form can also be characterized by other measures, such as fractal dimensions for urban boundaries, density gradients, and cluster analysis of built-up patches.

## 6. Conclusions

This study provides new insights into the urban expansion patterns and land use efficiency of Hanoi, Vientiane, and Phnom Penh between 2017 and 2024. The findings reveal a shared trajectory of horizontal growth driven primarily by the expansion of built-up land rather than population demand across all three capitals, despite significant spatial variation. Phnom Penh exhibits the most intensive form of land expansion, where the rate of land consumption substantially exceeds population growth, accompanied by high LUE and strong spatial clustering pattern in which similar LUE values are concentrated in contiguous areas. Vientiane shows rapid sprawl characterized by weak spatial cohesion, as urban development is fragmented and dispersed across space. In contrast, Hanoi, despite having the lowest LUE, maintains a relatively organized urban form, where development follows more continuous and planned spatial structures rather than scattered expansion. By integrating SDG 11.3.1 metrics with spatial autocorrelation and compactness analysis, this study demonstrates a robust framework for evaluating spatial efficiency, understood as the effectiveness of land use in relation to population distribution, in fast-growing urban contexts. These multidimensional results underscore how urban form, land use efficiency, and the degree of spatial concentration of development jointly shape urban growth trajectories, reflecting broader differences in planning institutions, land governance, and investment models.

The regional implications are clear that without strategic intervention, cities in the Indochina region risk further spatial fragmentation, unsustainable patterns of land consumption, and rising spatial inequality. The evidence presented reinforces the urgent need to reorient urban development toward compact, spatially coordinated, and inclusive growth strategies aligned with SDG 11. This approach offers a critical pathway for managing the urban transition in Southeast Asia in a more efficient, balanced, and sustainable manner.
